# Increased Institutional Surgical Experience in Robot-Assisted Radical Hysterectomy for Early Stage Cervical Cancer Reduces Recurrence Rate: Results from a Nationwide Study

**DOI:** 10.3390/jcm9113715

**Published:** 2020-11-19

**Authors:** Linnea Ekdahl, Emelie Wallin, Emilia Alfonzo, Petur Reynisson, Celine Lönnerfors, Pernilla Dahm-Kähler, Henrik Falconer, Jan Persson

**Affiliations:** 1 Department of Obstetrics and Gynaecology, Division of Gynaecologic Oncology, Skåne University Hospital, 22185 Lund, Sweden; Linnea.ekdahl@med.lu.se (L.E.); Petur.reynisson@med.lu.se (P.R.); Celine.lonnerfors@skane.se (C.L.); 2 Department of Clinical Sciences, Obstetrics and Gynaecology, Faculty of Medicine, Lund University, 22185 Lund, Sweden; 3 Department of Women’s and Children’s Health, Division of Neonatology, Obstetrics and Gynecology, Karolinska Institutet, 14186 Stockholm, Sweden; Emelie.wallin@sll.se (E.W.); Henrik.falconer@sll.se (H.F.); 4 Department of Pelvic Cancer, Karolinska University Hospital, 14186 Stockholm, Sweden; 5 Department of Obstetrics and Gynecology, Sahlgrenska University Hospital, 41345 Gothenburg, Sweden; Emilia.alfonzo@vgregion.se (E.A.); Pernilla.dahm-kahler@vgregion.se (P.D.-K.); 6 Institute of Clinical Sciences, Sahlgrenska Academy at University of Gothenburg, 41345 Gothenburg, Sweden

**Keywords:** cervical cancer, robotic radical hysterectomy, recurrence rate, learning curve

## Abstract

The aim of this study was to evaluate the impact of institutional surgical experience on recurrence following robotic radical hysterectomy (RRH) for early stage cervical cancer. All women in Sweden who underwent an RRH for stage IA2-IB1 cervical cancer at tertiary referral centers from its implementation in December 2005 until June 2017 were identified using a Swedish nationwide register and local hospital registers. Registry data were controlled by a chart review of all women. Recurrence rates and patterns of recurrence were compared between early and late (≤50 vs. >50 procedures) institutional series. Six hundred and thirty-five women were included. Regression analysis identified a lower risk of recurrence with increased experience but without a clear cut off level. Among the 489 women who did not receive adjuvant radio chemotherapy (RC-T), the rate of recurrence was 3.6% in the experienced cohort (>50 procedures) compared to 9.3% in the introductory cohort (*p* < 0.05). This was also seen in tumors < 2 cm regardless of RC-T (*p* < 0.05), whereas no difference in recurrence was seen when analyzing all women receiving RC-T. In conclusion, the rate of recurrence following RRH for early stage cervical cancer decreased with increased institutional surgical experience, in tumors < 2 cm and in women who did not receive adjuvant RC-T.

## 1. Introduction

Robotic radical hysterectomy (RRH) for early stage cervical cancer was introduced in Sweden in December 2005, gradually replacing open surgery as the primary surgical method. Concurrently, a rapid increase in robotic surgery in women with endometrial cancer occurred. In Sweden, the vast majority of the more than 3000 new cases of gynecological cancers annually are centralized to seven university tertiary centers with subspecialized surgeons. Approximately 550 women with novel cases of cervical cancer are diagnosed annually where approximately 65% of cases allow for primary surgery [1]. Preoperative evaluation, patient selection, principles for adjuvant radio chemotherapy (RC-T) and follow-up adhere to national guidelines [2].

Recent publications have raised concerns regarding the oncologic safety of RRH [3,4,5]). The randomized trial by Ramirez et al. (LACC study) and the observational study by Cusimano et al. mainly compared traditional laparoscopic surgery (84% and 89% of the minimally invasive surgery (MIS) groups, respectively) to open surgery, whereas the US register study by Melamed et al., in which 79.8% of MIS was performed robotically, was carried out during a robotic surgery introductory phase (2010–2013) in a low case load per institution setting. In contrast, a nationwide Swedish study including 864 consecutive women (236 ORH and 628 RRH) with cervical cancer operated between 2011 and 2017 and, where the major contributing centers had passed the introductory phase of RRH, did not demonstrate an inferior survival rate for RRH compared to open radical hysterectomy (ORH). Since tumor size and adjuvant treatment had a skewed distribution in the Swedish study, a propensity score model was used, accounting for age, grade, tumor size, lymph vascular space invasion (LVSI), lymph node status, primary treatment, and year of diagnosis and a similar oncologic outcome was demonstrated [6]. A Danish nationwide study of 1125 women did not find an increased risk of recurrence after the adoption of robotic radical hysterectomy [7]. In tumors less than 2 cm where the risk of recurrence is lower, a large amount of material is needed to investigate the recurrence rate. Existing studies have been unable to evaluate this subgroup properly [3,4,5,8]. Previous studies have shown a reduction in surgical time, blood loss and the rate of postoperative complications with increased surgical experience after 28–50 surgeries [9,10,11,12,13]. Two recent single institution studies, including 165 and 168 RRH, respectively, demonstrated reduced recurrence rates with increased experience [14,15]. The former used a multivariate risk-adjusted cumulative sum analysis and found a learning phase of 61 RRHs whereas the latter divided their experience based on the year of enrollment, which translated into 77 RRHs [14,15].

The primary aim of this nationwide study was to evaluate the effect of the institutional surgical experience of RRH for early stage cervical cancer on recurrence rates and patterns of recurrence. The secondary aims were to investigate the impact of institutional surgical experience on types of recurrences and perioperative complications.

## 2. Material and Method

All women in Sweden ≥ 18 years with a preoperative stage IA2-IB1 (FIGO 2009) with squamous adenocarcinoma or adenosquamous histology who underwent pelvic lymphadenectomy and an RRH according to Querleu–Morrow classification types B2 or C1 (or similar to the classification at the one clinic performing RRHs before 2008) from the first RRHs performed from December 2005 to June 2017 were included [16,17]. All RRHs were performed without the use of an intrauterine manipulator. Women converted to open surgery were included on an intention to treat basis. The women were identified by, and data retrieved from, the Swedish Quality Register of Gynecologic Cancer (SQRGC) and controlled by a review of local hospital registers to identify any missing women in the national quality register [6]. A full chart review was thereafter performed on all women by three of the authors (L.E, E.W and E.A) to control and harmonize the existing register data according to predefined common criteria regarding demographic information, age, body mass index (BMI) kg/m^2^, smoking status, tumor histology, FIGO stage, tumor grade, LVSI, tumor size, lymph node status in the pathology report, adjuvant treatment, all intraoperative and postoperative complications within 30 days, and time and site of recurrences within 24 months (which was the total follow up time in all women). Per institution, operations were chronologically numbered. Tumor size was defined as the largest diameter in a preoperative cone biopsy or hysterectomy specimens, hence representing the minimum size of the tumor. Women with a tumor size > 40 mm at final pathology, positive lymph nodes or women with margins of <5 mm (this included women with parametrial involvement) were recommended adjuvant RC-T. Neither depth invasion, LVSI nor grade were used as separate parameters influencing primary or postoperative treatment. Intraoperative complications (defined as a complications diagnosed and treated during primary surgery, or directly related to surgery but diagnosed postoperatively) and postoperative complications up to 30 days post-surgery were registered; the latter using the Clavien–Dindo classification [18]. Exclusion criteria were high-risk histology, FIGO 2009 stage <1A2 or >1B1, intraoperative abortion of the RRH in favor of RC-T, an unwillingness to receive recommended adjuvant treatment or loss to follow up within 24 months. All inhabitants in Sweden are assigned a unique personal identification number used for population registration and in health care. Health care for cancer is only provided by public hospitals. Hence, a woman was only lost to follow up if she emigrated abroad.

Preoperative examination included a computer tomography (CT) scan of the abdomen and thorax and pelvic magnetic resonance imaging (MRI). At follow-up, a clinical examination was performed at four to six months intervals. The criteria for offering adjuvant RC-T remained unchanged during the observation time. If a patient presented with symptoms indicating a recurrence, radiological examinations as indicated were performed followed by a biopsy for final diagnosis. Oncological outcome data were registered at 24 months, defined by the date of histological verification for all women. The recurrences were grouped into four categories: locoregional (vaginal vault or local pelvic recurrences), abdominal (port and/or intraabdominal recurrences), lymph nodes/distant (lymph nodes outside the pelvis or other distant recurrences), and multiple (multifocal recurrences).

The possible impact of surgical experience on oncologic outcome might be influenced by whether or not adjuvant RC-T was administered. As a result, the data set was split into two subgroups and analyzed accordingly. RRH was introduced at the first institution in 2005, whereas the sixth institution performed their first RRH in 2014, at which point the primary institution had performed more than 150 RRHs. Considering different baseline surgical and robotic skills, institutional recurrence rate depending on time of introduction was investigated.

The institutional review boards at Lund University (DNR 2008-663), the Karolinska Institute (DNR 2015-2140) and Gothenburg University (DNR 397-18) approved this study.

### Statistical Analyses

A logistic regression analysis was used to evaluate the effect of surgical order, center of treatment, the patient’s age, tumor size and tumor histology on the probability of recurrence occurring up to 24 months. The results were tested against a null hypothesis of an unimproved recurrence rate over time. As a potential effect of learning likely diminishes over time and eventually has no impact, a logistic regression model was constructed to compensate for such an effect (Appendix A). Both surgical order and center of treatment can be viewed as parameters representing skill. A potentially different baseline surgical and robotic skill between hospitals would likely impact the calibration of the effect of surgical order. For this reason, the logistic regression model was applied for all included hospitals as well as for the three centers of treatment with the earliest implementation and the highest number of performed RRHs (>100). In order to establish a suitable cut off level for comparison of the absolute recurrence rates between early and experienced cohorts, a model was constructed to evaluate any decrease over time in tumors of a median size (Appendix B). For comparison of clinical and recurrence data and potential skewness between early and experienced cohorts, the chi^2^ test was used.

For the logistic regression, data were entered into a Microsoft Excel data base, pseudo-anonymized and analyzed using the Python package Statsmodel Discrete Logic (version 0.11.1, Texas, USA) (Appendix C). For the remaining analyses, the SPSS version 12.0 statistical software was used (SPSS, Chicago, IL, USA). A *p*-value of less than 0.05 was considered significant in all statistical tests.

## 3. Results

**Figure 1:**
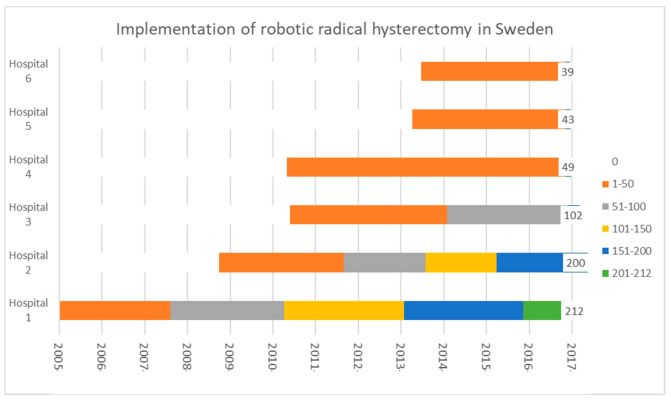
Implementation and number of robotic radical hysterectomies (RRHs) for stage IA2-IB1 (FIGO 2009) squamous, adenocarcinoma or adenosquamous cervical cancer performed per hospital in Sweden from the first RRH in December 2005 until June 2017.

Of the 719 identified women, 60 were excluded due to a high-risk histology (*n* = 20), FIGO 2009 stage <1A2 or >1B1 (*n* = 12), intraoperative abortion of the RRH in favor of RC-T (*n* = 17), an unwillingness to receive recommended adjuvant treatment (*n* = 6) or loss to follow up (*n* = 5, due to women who emigrated abroad). RRH was performed at nine institutions during the study period. Three hospitals performed ten or fewer RRHs, a number which was deemed unsuitable for statistical analysis, and were therefore excluded. The number of RRHs, included per site as well as distribution over time, can be seen in Figure 1.

**Figure 2:**
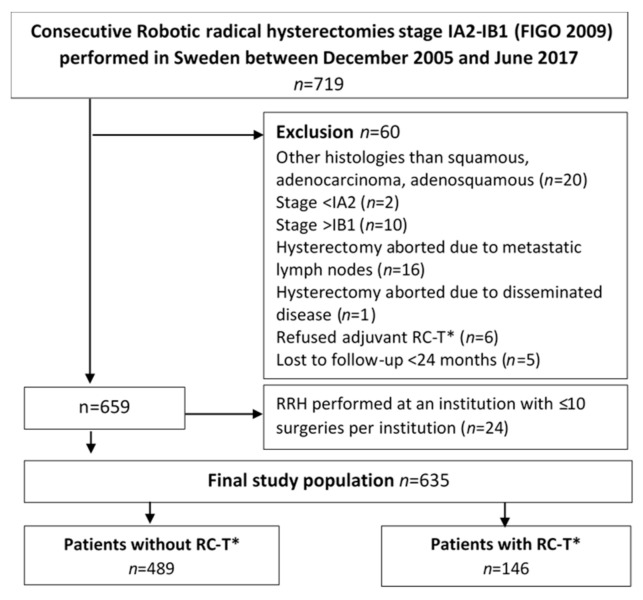
Strobe flow chart for all women in Sweden with stage IA2-IB1 squamous, adenocarcinoma or adenosquamous cervical cancer operated by robotic radical hysterectomy between December 2005 and June 2017 evaluating the impact of surgical experience on the rate of recurrence and postoperative complications. Sub-legend: * Oncologic protocol violations = women unwilling to receive recommended postoperative RC-T.

Of the 635 women included in the final analysis, 146 (23%) received adjuvant RC-T due to at least one of the following reasons (lymph node metastases (*n* = 68, 47%), tumor size > 40 mm at final histology (*n* = 11, 7.5%) or margins < 5 mm (*n* = 67, 45.5%). The remaining 489 women received surgery alone with RRH (Figure 2, strobe flow chart). Clinical and demographic data are shown in Table 1. Three hospitals performed > 100 RRHs and three hospitals < 50.

The regression analysis showed a decrease in the rate of recurrence with increased experience in women without RC-T for all six hospitals (*p* = 0.03) and for the three most experienced hospitals (*p* = 0.006). The statistical model using the three hospitals with >100 RRHs showed that the probability of recurrence decreased rapidly until about 50 surgeries, representing a probable inflection point (Appendix B). Therefore, when comparing the absolute recurrence rate in the whole study population, the first 50 cases from each of the six hospitals (introductory cohort) were compared with the remaining > 50 cases from the three most experienced hospitals (experienced cohort).

Of the 489 women with no RC-T, fewer recurrences occurred in the experienced cohort compared to the introductory cohort (3.6% compared with 9.3%, *p =* 0.009). In tumors < 2 cm, this was true both for tumors < 2 cm without RC-T (*n* = 373, 1.9% compared with 7.0%, *p* = 0.01) and tumors < 2cm regardless of adjuvant treatment (*n* = 43, 2.9% compared with 7.9%, *p* = 0.02). Extrapelvic (abdominal, multiple or nodal/distant) recurrences were seen predominantly in the introductory cohort (6 of 214 vs. 2 of 275) but the regression analysis did not verify a significant decline with experience for these few incidents (*p* =0.10). No difference with experience in overall recurrence or pattern of recurrence was seen in tumors ≥ 2 cm (16.1% vs. 10.0%, *p* = 0.33) or in women who received adjuvant RC-T (Table 1).

Of the 635 women, ten (1.6%) experienced an intraoperative complication and three conversions to laparotomy (0.5%) were necessary due to adhesions (*n* = 1), vessel injury (*n* = 1) and subcutaneous emphysema (*n* = 1). Almost 90% of the postoperatively (<30 days) diagnosed complications were mild or moderate (grade I-II) whereas injury to the ureter (*n* = 10), intraabdominal abscess (*n* = 7), port-hernia (*n* = 4), vesicovaginal fistula (*n* = 2), postoperative bleeding (*n* = 1), vaginal dehiscence (*n* = 1) and compartment syndrome of the legs (*n* = 1) occurred in 4.1%. The rate of postoperatively diagnosed complications (≥grade IIIa) decreased with increased experience (2.5% vs. 6.1%, *p* = 0.03) (Table 2). A significant decrease in complications directly associated with surgery was seen when these postoperatively diagnosed complications, i.e., ureter injury, vesicovaginal fistulas and compartment syndrome were added to the intraoperative complications group (*p* = 0.01). This second categorization was used due to the shortcoming of the Clavien–Dindo classification that does not classify intraoperative complications as a separate entity and where postoperatively discovered intraoperative complications are classified as postoperative.

## 4. Discussion

The rate of recurrence following RRH for early stage cervical cancer decreased with increased institutional surgical experience in women who did not receive adjuvant RC-T as well as in women with tumors < 2 cm, regardless of given adjuvant treatment. A similar decrease in recurrence was not seen in women with tumors ≥ 2 cm. In women with tumors ≥ 2 cm or who received adjuvant RC-T, the inherent higher risk of extrapelvic recurrence, the possibility of occult disease at the time of surgery, and probable prevention of locoregional recurrence following RC-T rather than the surgical technique per se, are probable contributing factors. The study is, however, underpowered for smaller subgroup analyses. But we cannot exclude that increased surgical experiences have less positive impacts on women with larger tumors where no RC-T is administered. For all women having undergone RRH, surgical complications were less frequent in the experienced cohort.

Previous studies have demonstrated a positive effect of increased experience with RRH in regard to surgical time, blood loss, and early postoperative complications, which was also seen in the present study [9,10,11,12]. A positive impact on oncological outcome with increased experience has been shown for robotic radical prostatectomy by Galfano et al. and was implied by Chong et al., who investigated RRHs during the learning phase compared to conventional laparoscopic radical hysterectomies performed by experienced surgeons. [19,20]. Two recent single-institution studies investigated the impact of learning curve on oncological outcome following RRHs for early stage cervical cancer and found improved survival rates with increased surgical experience, achieving similar levels of adequate experience to our study [14,15]. Neither of the studies discussed or further clarified which elements associated with increased experience would be expected to have a positive effect on recurrence rates. Rather than discarding RRHs for early stage cervical cancer, the authors similar to our experience, emphasize the necessity of centralized health care combined with a validated learning curriculum to shorten and make the learning process more effective. In addition, taking into account the institutional oncological outcomes when counseling patients is emphasized [14,15]. The results from these two studies and the present nationwide study might explain the discrepancy in the recurrence rate following RRH and open radical hysterectomy (ORH) in the US register study by Melamed et al. and the Swedish study by Alfonzo et al., both national studies including 2461 (978 RRH) and 864 women, respectively [3,6]. The increased rate of recurrence after RRH compared to ORH in the former was probably partly due to a low case load setting, including data from 479 institutions and with 357 institutions sharing a total of 978 RRHs over the studied period (personal communication Dr Melamed) representing an introductory phase of robotic surgery [3,6]. Alfonzo et al. on the other hand found no difference in recurrence rate for RRHs and ORHs performed between 2011 and 2017 when the two major contributing institutions had passed their learning phase [6]. As described in the introduction, confounding factors were compensated for using propensity score analysis. A similar nationwide study from Denmark, where the organization of care is similar to Sweden, also failed to show differences in recurrence in ORH and RRH groups. These studies represent hybrids between prospectively retrieved quality register data and retrospective control of these data. According to a post-hoc 80% power analysis of 236 ORH and 628 RRH included in the Swedish study, a difference in recurrence of up to 5.7% for either group (compared with 9.5% in the LACC study) theoretically may have remained unnoticed. However, we believe it is unlikely that a difference in recurrence in the magnitude of what is demonstrated by the LACC study would have been missed.

In Sweden, the centralization within gynecological cancer surgery, adherence to national guidelines as well as strict requirements/curriculum for achieving a subspecialization in gynecological cancer surgery (at least 4 years at a tertiary unit) ensures conformity. The requirements for tertiary units providing subspecialization and the credentials for subspecialization are defined by the Swedish Society of Obstetrics and Gynaecology. Surgery within gynecologic oncology is, with few exceptions, performed at tertiary referral centers. Within these centers, robotic gynecological cancer surgery, including RRH, is performed by a limited number (1–3 per institution) of surgeons to ensure an adequate case load per surgeon and to further enhance quality, the bedside assistant is usually also an experienced robotic surgeon. Even though the number of RRHs per institution per year, despite centralization, were relatively low (between 7 and 23 in 2017) the six included university hospitals had an annual case load of between 66 and 302 robotic; mainly gynecological cancer procedures (in 2017). It is probable that training by, and exchange of experience with surgeons already experienced in RRH, may affect baseline skills for institutions with a later implementation of RRH. This was implied when comparing early cohorts from the hospitals where RRH was firstly implemented to the two hospitals with the latest introduction (Figure 1 and Table S1 Supplementary Material). A further indication of interinstitutional exchange of experience was seen in the regression analysis where a stronger significance level was present when comparing the effect of learning for the two hospitals with the latest introduction to all six hospitals.

Overall organization of care, including case load per surgeon of RRH and other robotic procedures, and timing of the study in relation to implementation of a novel technique, must be taken into consideration when comparing a new approach to a well-established surgical method. This is further emphasized by Doo et al. and Sert et al. where the former reported a higher risk of recurrence following RRH compared to ORH during an introductory phase, whereas Sert et al. found no difference in their multicenter study with a higher annual case load per institution [8,21]. Although the ORH group in the LACC trial and ORH group in the trial by Sert et al. were almost identical in terms of inclusion period, proportion of lymph node metastases, adjuvant treatment and follow-up time, there was, for unknown reasons, a substantial discrepancy in the oncologic outcome in favor of the ORH in the LACC study [4,21].

Recent studies have highlighted the importance of evaluating possible factors inherent to robotic and laparoscopic surgery that might influence oncologic safety of the procedure, and potential areas for improvement and learning [4,22,23]. A possible contributing factor as suggested by Ramirez et al. in the LACC trial is the use of an intrauterine manipulator, a device never utilized for RRH in Sweden. Instead a fornix-presenter (a simple tube or cup delineating the fornices) was used. A recent multi-institutional (89 centers) retrospective study (the SUCCOR study, including 291 MIS radical hysterectomies (RHs) of which 63 were robotic) comparing ORH and MIS, found MIS and the use of an intrauterine manipulator to be associated with an increased risk for recurrence. Given the low average institutional number of MIS RHs in general and RRHs in particular, this study may support our conclusion of the importance of experience [24]. Moreover, avoiding tumor contamination of the abdominal cavity during the opening of the vagina and the retrieval of the specimen might be of importance. Köhler et al. recommended an initial closure of the vagina to prevent this exposure [25]. The very low incidence of lymph node metastases in the Köhler study (3%) compared to the present study (10.7%) makes a direct comparison impossible. Although vaginal closure was not applied in this material an increased awareness and preventive measures regarding this possible risk factor might theoretically have influenced our results. Alternatively, a large cone biopsy at upfront surgery to remove an exposed tumor may be applied. Another possible risk factor associated with robotic surgery is overestimation of distance due to magnification. This could lead to a larger proportion of women undergoing RRH having surgical margins close to insufficient. The fixed grip force of the instruments that might crush metastatic lymph nodes if directly grasped and cause an inadvertent tumor spread would only be of importance if the nodes were metastatic. The performance of an adequate sentinel node technique and the extent of the pelvic lymph node dissection, measures to prevent contamination of tumor as well as the surgical margins are possible areas where surgical experience would have a positive impact, which might influence the oncological outcome for the patient. Previous studies on surgical experience in robotic surgery have focused on surgical time and rate of complications and have unsurprisingly shown a decrease with time. [9,10,11,12]. The use of CO_2_ and pneumoperitoneum have been suggested to have a negative oncologic effect, although neither can explain the reduced recurrence rate with increased experience observed in the study.

In Sweden, RRHs were first implemented in 2005. Later implementations were aided by study visits and proctoring by surgeons from either of the two institutions with the earliest start ensuring a homogenous surgical approach among all centers. RRHs rapidly became the primary approach of choice, initially limited by robot access at some institutions. Laparoscopic RHs were never implemented in Sweden.

Our decision to compare institutional experience rather than individual surgeons’ experience was due to the rarity of the procedure and the fact that some surgeons retired or stopped performing RRHs during the 12-year study period. This is a weakness of the study. However, within each institution, one surgeon performed the procedure from implementation, representing continuity. During 2017, either one of two surgeons at each of the two main contributing centers performed all RRHs. The institutional experience also entails the experience of the whole surgical team, which is necessary for a successful robotic program. Surgeons introduced at a later date likely benefited from the experience of the novel surgeon in terms of surgical time and rate of complications. The present study suggests that this transfers into an oncological benefit. To what extent an increased individual surgical experience, transfer of experience or improvements of team performance affect results remains unclear. This is a potential weakness of our study but also an incentive to evaluate institutional performance rather than individual surgeons’ results. The effect of a difference in baseline skill is discussed and compensated for in the statistical analysis.

The heterogeneity of cancer and its inherent characteristics and risk factors as well as the multifactorial aspects of learning and experience does not allow for an exact cut off level when experience with an impact on oncological outcome has been achieved. The level utilized in the present study is within the range previously reported by other authors.

The strengths of the study are the nationwide setting with consecutive procedures with only five women (0.7%) lost to follow up and the quality and conformity of data secured by a chart review using commonly defined criteria for clinical parameters of all women performed by three of the investigators with regular audits. The organization of care with centralization both within gynecological cancer surgery and gynecological oncology allowed for an investigation of the nationwide implementation of RRH as well as a true representation of the rate of recurrence. Utilizing recurrence rate at 24 months ensures the same follow-up for all included women although excludes the recurrences that occur at a later date. Consequently, a weakness of the study is that a direct comparison with similar cohorts with longer follow-up is not possible.

Another potential weakness is that data on LVSI and grade were missing for approximately 1/3 of women. However, the new 2020 WHO histological classification of tumors of the cervix does not include grade for squamous cell carcinomas. Furthermore, the prognostic value of grade is debated when newer classifications are suggested [26]. According to Swedish national guidelines, these parameters do not influence selection for surgery or postoperative RC-T. In the majority of women where these data are available, there is no difference between early and late groups. Furthermore, it is unlikely that the proportions of these parameters were differently distributed throughout the country or over time. Therefore, we do not believe that the lack of data on these parameters in some women affect the interpretation of our results.

In the light of the available evidence, the question arises whether a possible increased risk of recurrence can be accepted even when potentially hazardous parts of an RRH can be compensated for. A more accurate detection of sentinel lymph nodes with robotic surgery might decrease the rate of undetected lymph node metastases and facilitate a structured and safely implemented sentinel lymph node (SLN) concept, thereby minimizing the risk of lower limb lymphedema, a major lifelong side effect in some women [27]. In obese women, MIS in endometrial cancer favors immediate and long-term wound healing and reduces infections [28]. Bowel obstruction and intraabdominal adhesions are less common following MIS, the latter is especially beneficial when adjuvant RC-T is indicated [29,30,31,32]. Finally, future technological progress and development including tracers, intraoperative tumor markers and intraoperative imaging will likely be dependent on a minimally invasive platform. Randomized trials investigating the optimal surgical approach for cervical cancer and the future of RRH are currently ongoing [33,34].

Although recent studies have led to a change in practice patterns at many institutions where MIS for cervical cancer has been abandoned in favor of ORH, the results are conflicting. The reduced rate of recurrence and rate of serious postoperative complications, as well as the reduced rate of multiple and intraabdominal recurrences following RRH for early stage cervical cancer with increased surgical experience, must be taken into account when organizing care and counseling the patient prior to surgery. This is supported by a previous Swedish nationwide study as well as two recent publications investigating the influence of learning curve where similar recurrence rates for RRH and ORH were seen at high-volume centers after the implementation period [6,14,15]. When interpreting available studies and performing future studies on RRH for cervical cancer, the negative impact of novel early adopters and low-volume surgeons on the rate of recurrence and postoperative complications must be considered.

## 5. Conclusions

The rate of recurrence following RRH for early stage cervical cancer decreased significantly with increased institutional surgical experience in the larger subgroup of women who did not receive adjuvant RC-T as well as in women with tumors <2 cm, regardless of the given adjuvant treatment.

Studies on RRH for cervical cancer, and organization of care, should consider the negative impact of early adopters and low volume surgeons on the rate of recurrence. A multicenter RCT, which started in 2019, comparing ORH with RRH (the RACC study) where bias by early adoption and low case load is minimized, is currently ongoing [33].

## Figures and Tables

**Table 1:** Characteristics of 635 patients with stage IA2-IB1 squamous, adenocarcinoma or adenosquamous cervical cancer operated with robotic radical hysterectomy in Sweden between December 2005 and June 2017 with or without postoperative radio chemotherapy. Introductory and experienced cohort refer to the first 50 surgeries per institution compared to all following robotic radical hysterectomies.

Total (*n* = 635)		No Radio Chemotherapy (*n* = 489)			Radio Chemotherapy (*n* = 146)		
		Introductory cohort		Experienced cohort	Introductory cohort		Experienced cohort
median (range)/number (%)		≤50 *n* = 214	* p * -value	>50 *n* = 275	≤50 *n* = 66	* p * -value	>50 *n* = 80
Age	42.9 (22.3–86.6)	42.0 (23.8–86.6)	*p* = 0.25 ᵃ	42.8 (22.3–83.0)	47.3 (25.3–83.2)	*p* = 0.77 ᵃ	46.1 (24.9–79.9)
BMI ^1^	25 (17–59.9)	25.1 (17.6–59.9)	*p* = 0.52 ᵃ	24.5 (17.0–48.2)	24.7 (19.0–35.0)	*p* = 0.98 ᵃ	26.1 (17–38.9)
Smoking *	Yes No Unknown **	46 (31.7%) 99 (68.3%) 69(32.3%)	* p * = 0.25	81 (37.7%) 134 (62.3%) 60 (21.8%)	14 (33.3%) 28 (66.7%) 24 (36.4%)	*p* = 0.59	25 (38.5%) 40 (61.5%) 15 (18.8%)
	
			*p* < 0.01			*p* = 0.02	
Tumor size	13 (0.2–62)	11.0 (1.5–60)	*p* = 0.43 ᵃ	10 (0.2–62.0)	20 (3–48)	*p* = 0.08 ᵃ	22.5 (2–50)
Figo Stage IA2	71 (11.2%)	26 (12.1%)		43 (15.6%)	0 (0.0%)		2 (2.5%)
Figo Stage IB1	564 (88.8%)	188 (87.9%)	*p* = 0.27 ᵇ	232 (84.4%)	66 (100.0%)	*p* = 0.68 ᵇ	78 (97.5%)
Histology							
Squamous	367 (57.8%)	116 (54.2%)		152 (55.3%)	43 (65.2%)		56 (70%)
Adenocarcinoma	233 (36.7%)	83 (38.8%)	*p* = 0.24 ᵇ	113 (41.1%)	18 (27.2%)	*p* = 0.82 ᵇ	19 (23.7%)
Adenosquamous	35 (5.5%)	15 (7.0%)		10 (3.6%)	5 (7.6%)		5 (6.3%)
LVSI *	Yes No Unknown **	38 (26.6%) 105 (73.4%) 71 (33.2%)	*p* = 0.64	46 (34.3%) 143 (75.6%) 86 (31.3%)	39 (75.0%) 13 (25.0%) 14 (21.2%)	*p* = 0.65	42 (71.2%) 17 (28.8%) 21 (26.2%)
	
			*p =* 0.65			*p* = 0.69	
Grade *	1 + 2 3 Unknown **	107 (63.7%) 61 (36.3%) 46 (21.59)	* p * = 0.60	82 (60.7%) 53 (39.3%) 140 (50.8%)	31(53.4%) 27 (46.6%) 8 (12.1%)	* p * = 0.71	27 (52.9%) 24 (47.1%) 29 (36.3%)
	
			*p* = 0.001			*p* = 0.06	
Reason for adjuvant treatment							
Metastatic nodes					33 (50.0%) 6 (9.1%) 27 (40.9%)	*p* = 0.51 ᵇ	35 (43.7%) 5 (6.3%) 40 (50.0%)
Tumor > 40 mm							
Insufficient margins							
Recurrence ≤ 24 months	51 (8.0%)	20 (9.3%)	*p* = 0.01 ᵇ	10 (3.6%)	9(13.6%)	*p* = 0.82 ᵇ	12(15%)
Recurrence rate ≤ 24 months in tumors < 2 cm	22/431 (5.1%)	11/158 (7.0%)	*p* = 0.01 ᵇ	4/215 (1.9%)	4/31 (12.9%) ^2^	*p* = 0.83 ᵇ	3/27 (11.1%) ^2^
Recurrence rate ≤ 24 months in tumors ≥ 2 cm	29/204(14.2%)	9/56 (16.1%)	*p* = 0.33 ᵇ	6/60 (10.0%)	5/35 (14.3%) **	*p* = 0.74 ᵇ	9/53 (17.0%) **
		**All patients’ tumors < 2 cm** **with and without RC-T**			**All patients’ tumors ≥ 2 cm** **with and without RC-T**		
		Introductory cohort	*p*-value	Experienced cohort	Introductory cohort	*p*-value	Experienced cohort
Recurrence rate ≤ 24 months		15/189 (7.9%)	0.02 ᵇ	7/242 (2.9%)	14/91 (15.4%)	0.67 ᵇ	15/113 (13.3%)

Sub-legend: * Percentages refer to women with known information. ** Percentages refer to all women within that group. ^1^ Data unavailable in 17 women. ^2^ No significant difference in rate of node positive women between the introductory and experienced cohort. ᵃ Mann-Whitney test. ᵇ Chi-squared test.

**Table 2:** Number and percentage of intraoperative and postoperative complications according to Clavien–Dindo in the introductory and experienced surgical cohort of robotic radical hysterectomy with and without radio chemotherapy in Sweden between December 2005 and June 2017.

*n* (%) Total *n* = 635	Total *n* = 635	Introductory Cohort ≤50 *n* = 280	Experienced Cohort >50 *n* = 355	*p*-Value
Postoperative complications Grade I-IIIb	186 (29.3%)	90 (32.1%)	96 (27.0%)	*p* = 0.20 ᵃ
Postoperative complications Grade ≥ IIIa	26 (4.1%)	17 (6.1%)	9 (2.5%)	*p* = 0.03 ᵃ
Intra-operatively diagnosed complications	10 (1.6%)	7 (2.5%)	3 (0.8%)	*p* = 0.10 ᵃ
Intra-operative and surgical postoperative complications ᵇ	23 (3.6%)	16 (5.7%)	7 (2.0%)	*p* = 0.01 ᵃ

Sub-legend: ᵃ Chi-squared test. ᵇ Combination of complications discovered intraoperatively and surgical complications discovered postoperatively i.e., injury to the ureter, compartment syndrome and vesicovaginal fistula.

**Figure A1:**
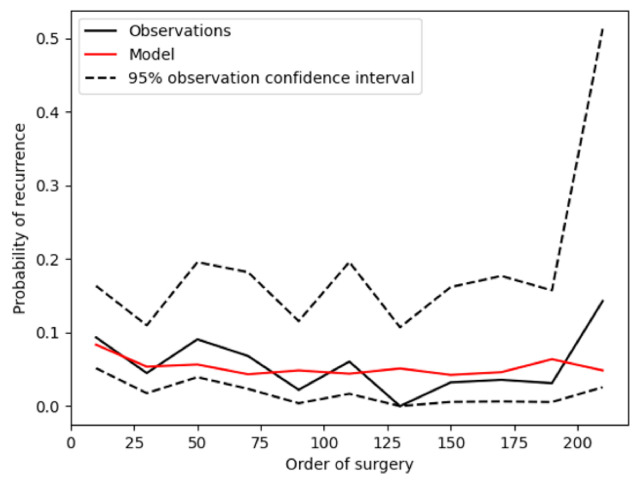
Analysis performed on all hospitals.

**Figure A2:**
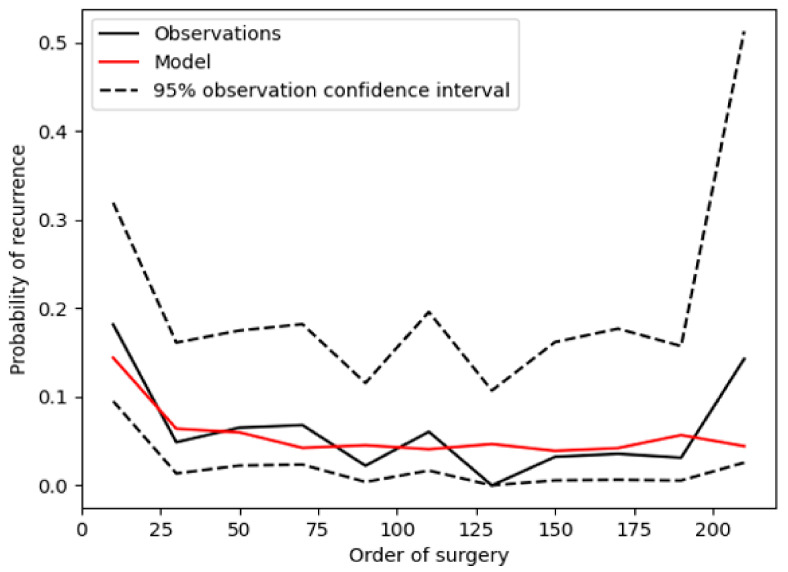
Analysis performed on the three hospitals with the earliest implementation and >100 RRH.

**Figure A3:**
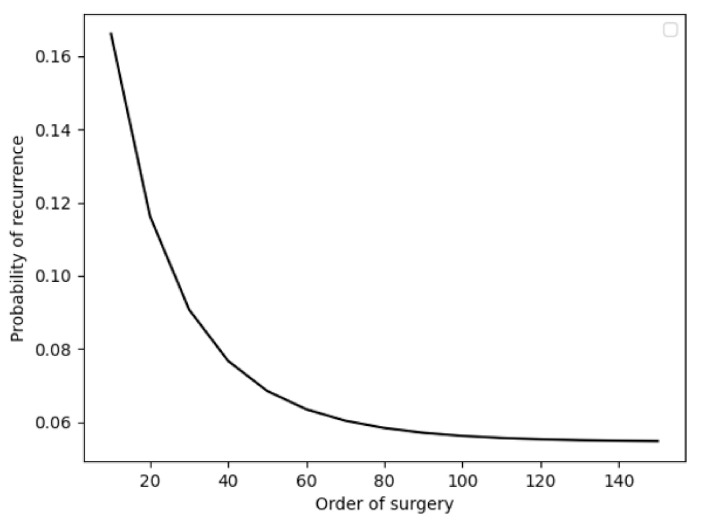
Constructed model showing the probability of recurrence given the order of surgery for a median tumor size of 21 mm (median in sample) for the model using the three hospitals with >100 RRH.

## Supplementary Materials

The following are available online at https://www.mdpi.com/2077-0383/9/11/3715/s1, Table S1: Number of robotic surgeries per hospital during the introduction of robot assisted radical hysterectomy (RRH) (≤50 surgeries) and the experienced years (>50 surgeries). Rate of recurrence and tumor size in the cohort without Radio chemotherapy (RC-T) presented per hospital.

## Appendix A

The connection between the probability of recurrence within 24 months and the variables surgical order, hospital, patient age, tumor size and histology are evaluated using logistic regression. Since it is reasonable to believe that the impact of the skill component decreases over time and eventually goes away, the surgical order has been transformed. The resulting model can be seen below.

$$\log\left(\;\frac{p}{1-p}\;\right)=\;\;\alpha_{\mathrm{size}}\;\cdot \;\mathrm{size}\;+\;\alpha_{\mathrm{order}}\left(1-\mu^{\mathrm{order}}\right)\;+\;\overset{\mathrm{Hospitals}}{\underset{h}{\sum }}\alpha_{h}\;+\;\overset{\mathrm{Tumor}\;\mathrm{types}}{\underset{t}{\sum }}\alpha_{t}.$$

Both surgical order and hospital can be viewed as parameters representing skill. The hospitals have performed varied number of surgeries, ranging between 39 and 212. If their baseline skills are different this is likely to impact the calibration of the effect of surgical order. Therefore, it is important to either include both the hospital specific parameters, or to perform the analysis only on hospitals with a matching number of surgeries. Due to the interpretability as well as the repeatability of the results, the transformation parameter *µ* = 0:96 was estimated using maximum likelihood prior to performing the standard logistic regression using the python package statsmodels.discrete.discrete model in Logit (version 0.11.1). Since it is reasonable to believe that the impact of surgical order is highest if the patient does not undergo adjuvant treatment, the data set is split in two and the analysis is performed on the group without adjuvant treatment.

Comparing modelled probabilities to the observed fraction of recurrence within 24 months in women without adjuvant radiochemotherapy. The observed fractions were estimated using an average of ten consecutive surgeries. The confidence intervals were calculated using Wilson’s method. The steep rise at the end is due to one recurrence in the last ten patients.

Comparing modelled probabilities to the observed fraction of recurrence within 24 months. The observed fractions were estimated using an average of ten consecutive surgeries. The confidence intervals were calculated using Wilson’s method. The steep rise at the end is due to one recurrence in the very last ten patients.

**Table A1:** Connection between the probability of recurrence within 24 months and the variables tumor size and surgical order.

	*p*-Value
Tumor size at all hospitals	0.001
Order of surgery at all hospitals	0.028
Tumor size at three largest hospitals	0.006
Order of surgery at three largest hospitals	0.006

## Appendix B

Before 20 and after 80 there is little difference in probability. Therefore, a cut off of 50 procedures was used.

## Appendix C

Copyright for statsmodels.discrete.discrete_model.Logit (version 0.11.1) Copyright (C) 2006, Jonathan E. Taylor. All rights reserved. Copyright (c) 2006–2008 Scipy Developers. All rights reserved. Copyright (c) 2009–2018 statsmodels Developers. All rights reserved.

Redistribution and use in source and binary forms, with or without modification, are permitted provided that the following conditions are met: a. Redistributions of source code must retain the above copyright notice, this list of conditions and the following disclaimer. b. Redistributions in binary form must reproduce the above copyright notice, this list of conditions and the following disclaimer in the documentation and/or other materials provided with the distribution. c. Neither the name of statsmodels nor the names of its contributors may be used to endorse or promote products derived from this software without specific prior written permission.

This software is provided by the copyright holders and contributors “as is” and any express or implied warranties, including, but not limited to, the implied warranties of merchantability and fitness for a particular purpose are disclaimed. In no event shall statsmodels or contributors be liable for any direct, indirect, incidental, special, exemplary, or consequential damages (including, but not limited to, procurement of substitute goods or services; loss of use, data, or profits; or business interruption) however caused and on any theory of liability, whether in contract, strict liability, or tort (including negligence or otherwise) arising in any way out of the use of this software, even if advised of the possibility of such damage.
