# Supplementary material for: Increased Institutional Surgical Experience in Robot-Assisted Radical Hysterectomy for Early Stage Cervical Cancer Reduces Recurrence Rate: Results from a Nationwide Study
Source: J Clin Med. 2020 Nov 19;9(11):3715. doi: 10.3390/jcm9113715 (PMC7699339; doi:10.3390/jcm9113715)
Supplement: Supplementary file 1 [file jcm-09-03715-s001.pdf]

Table A Supplementary material. Number of robotic surgeries per hospital during the introduction of robot assisted radical hysterectomy (RRH) (≤50 surgeries) and the experienced years (>50 surgeries). Rate of recurrence and tumor size in the cohort without Radio chemotherapy (RC-T) presented per hospital.

**University Hospital**	**Hospital 1**	**Hospital 2**	**Hospital 3**	**Hospital 4**	**Hospital 5**	**Hospital 6**
Year of onset of RRH	2005	2009	2011	2010	2014	2014
Average annual volume of RRH* during the years of introduction ≤50 surgeries	16	16	13	10	12	10
Average annual volume of total robotic surgery during the years of introduction ≤50 surgeries	106	122	51	104	86	65
Average annual volume of total robotic surgery during the experienced years >50 surgeries	265	243	74	-	-	-
**RRH without RC-T**						
Recurrence rate introductory cohort ≤50 surgeries	8.6%	20%	10%	10.5%	2.6%	3.6%
Median tumor size introductory cohort ≤50 surgeries	10	15	10.5	11.5	12.5	10.8
Recurrence rate experienced cohort >50 surgeries	2.6%	5%	2.5%	None performed	None performed	None performed
Median tumor size experienced cohort >50 surgeries	10	10	9.5			

*does not include RRH for high risk histology cervical cancer or uterine stage II endometrial cancer.
